# Monthly Summary

**Published:** 1871-01

**Authors:** 


### MONTHLY SUMMARY.

---

*Bad Effects of Chloral.*—A patient in Guy's Hospital, with aneurism of the thoracic aorta, being given half a drachm of chloral to allay pain and procure sleep, became unconscious immediately on the administration of the dose, with lividity and coldness of face and hands, and respiration only at long intervals, death seeming imminent for five hours. These symptoms passed off during the following day. In commenting on the case, Dr. Habershon remarked that it confirmed the opinion he had formed from observation of cases of pneumonia and bronchitis; namely, that chloral tends to cause bronchial and pulmonary congestion, through its action on the pneumogastric nerve, and it should not be given where embarrassment to respiration is liable to occur.

Dr. R. C. Shettle, Physician to the Royal Berkshire Hospital, in the course of an address before the Pathological Society of Reading, after referring to Dr. Richardson's statement that under the influence of chloral formiate of soda is formed, and the coagulating power of the blood is much diminished, adds that "it is very evident that not only is passive hæmorrhage from an open vessel favored by the administration of chloral, but from the tendency which exists from the continuous administration of the drug to produce decomposition of the blood, a small loss of blood would be very possibly followed by serious symptoms. Moreover, it can scarcely be a question that the tolerance of the drug is much greater in some individuals than in others; and possibly the same person at different times might bear its administration very differently." If these views be correct, much caution would seem to be called for in using chloral in midwifery cases.—*Medical Gazette.*

*Treatment of Chilblains*—For these exceedingly obstinate and troublesome affections, the *Medical Press and Circular* recommends the following formula:—Liniment of belladonna, two drachms; liniment of aconite, one drachm; carbolic acid, ten drops; collodion flexile, one ounce; to be applied with a camel's hair brush. If the chiblains vesicate, ulcerate, or slough, the aconite should be omitted.

---

*Vaccination in the Negro.*—Dr. Mortimer, an English Naval Surgeon, states, as the result of his experience in a number of cases, that the vaccine vesicle in the negro, does not develope until from the eleventh to the thirteenth day. Will some of our Southern readers inform us if they have noticed the same phenomenon; and also if admixture of white blood have any modifying influence?—*Medical Gazette.*

---

*Death from Chloroform.*—Dr. E. C. Hamilton of Washington, Ohio, writes to the "Dental Register" as follows:

" Mrs. Garris, the wife of our county sheriff, came to my office on Thursday afternoon for the purpose of having three roots and one molar tooth extracted. Dr. I. G. Wilson, a physician of this place, was with her, to administer chloroform. After I examined the mouth, and laid out the necessary forceps for the operation, Dr. Wilson proceeded with the chloroform, and after administering probably ten minutes(he did not intend to put her entirely under its influence), he called to me to take out one tooth. I then extracted the root of the right superior lateral incisor, only about one-fourth of an inch in length, and attended with no difficulty whatever. The patient then asked for more chloroform, and the Doctor was on the point of giving it, when death ensued. Three physicians worked probably three hours to restore life, but all in vain.

"These are the facts of the case." Three of our medical gentlemen, who use chloroform extensively, attempted to charge upon 'the shock of extraction' the cause of death, but the people so ridiculed the idea that they abandoned it. Of course all who are acquainted with the facts attach no blame or responsibility upon me, as it is well known that I have invariably refused to use chloroform, and have advised in every case against its use in dental surgery.

"I make this statement to you so that you may know what the facts are, and not be misled by newspaper statements, in case you desire to refer to the case in your journal.

---

*Influence of the Pancreas on Fat.*—According to Dr. Dobell, "the influence exerted by the pancreas upon fats appears to be breaking up the aggregation of the crystals of the fat, and alters its hydration. It alters the molecular condition of the fat mingling it with water in such a way that even ether cannot separate the fat from the water. A permanent emulsion is thus formed ready to mix with a larger quantity of water whenever it may be added. The pancreas, therefore, in acting upon fat, does not decompose it into fatty acid and glycerine; the absence of glycerine from the watery stratum and the presence of the glycerine in the pancreatized fat of the ethereal stratum having been demonstrated. It is well known that, in addition to the influence of the pancreas upon fat, it has the power of converting starch into glycose by simple mixture. This property remains to a certain extent after the pancreas has exhausted its property of acting upon fat. The quantity of pancreas that before mixture with fat will convert about eight parts of starch into glycose, after saturation with fat will still convert about two parts of starch into glycose.—*Proceedings of the Royal Society.*

---

*Manufacture of Ozone.*—Mr. Loew, of New York, has discovered that ozone is formed in rapid combustion. The German chemist, Carl Thaur, finds the observations of Mr. Loew to be correct.

A small quantity of ozone is always found in that part of the air which is immediately in contact with the lower part of the hydrogen flame, and its presence can be shown by drawing the air through a glass tube. The point of the tube ought to be inserted into the lower half of the flame, and the draft must be strong enough to divert the flame a little from the perpendicular, but not enough to draw the unconsumed gases through it, as they at once destroy the ozone. Burning charcoal yields no ozone, for the reason that the carbon absorbs both atoms of oxygen to produce carbonic acid. This method of the formation of ozone is of great interest, and may eventually lead to its practical application in bleaching and disinfecting. It would appear to be a cheaper and a better way to evolve the active oxygen than by electricity.—*Medical and Surgical Reporter.*

---

*Theory of Sleep.*—Dr. E. Sommer, in *Zeitschrift fur Rationelle Medicin,* declares that sleep is the result of a *deoxygenation* of the organism. "The blood and the tissues possess the property of storing up the oxygen inhaled, and then supplying it in proportion to the requirements of economy. When this state of oxygen is exhausted, or even becomes too small, it no longer suffices to sustain the vital activity of the organs, the brain, nervous system, muscles, &c., and the body falls into that particular state which we call sleep. During the continuance of this deep repose, fresh quantities of oxygen are being stored up in the blood, to act as a supply to the awakened vital powers. Rest produces, though in a less degree, the same effect as sleep in reducing the expenditure of oxygen."—*National Med. Journal.*

---

*The Physiological Effect of Muscular Exercise.*—In some remarks on this subject recently made before a medical society in New York, Dr, Austin Flint, Jr., stated that there is an increased quantity of excrementitious matter produced in the system on violent muscular exertion.

Some recent experiments seem to throw doubt on this statement, but he thought their results were not correct.

Within physiological limits, the elimination of this excess of effete matter is attended, if proper nutriment be supplied, by increased nutrition. If a man in health, eating and drinking according to a correct appetite, exercises his muscular system so as to increase to its highest point the elimination of effete matter, he will correspondingly increase the nutrition of his muscular system. If we can thus develope the muscular system the rest of the body will share in the improvement.

Can a man by such exercise successfully combat the tendency to deposits in his tissues, to the formation of tubercles?

Dr. F. thought such was possible ; he had seen consumptives, with tubercles already formed, go to the gymnasium and become hale and robust. He knew one case in particular where the patient became an accomplished gymnast after tubercles had been clearly diagnosed in his lungs. It would seem that these men might by increasing the deposit of normal material, have removed the disposition to the deposit of that which is abnormal.

Let a man moderately healthy work as hard as he pleases with his brain—if only he take the appropriate exercise to keep the system throwing off the effete matter, and appropriating in its place, healthy nitrogenized functioning matter, he will be comparatively safe.

It is better by constant exercise to keep ourselves healthy, than to break down every few months and have to take a vacation to recruit again.—*N. Y. Medical Journal.*

---

*A New Antiseptic.*—The hydrate chloride of aluminium, to which Mr. John Gambee has recently drawn the attention of medical men and the general public, appears to be a valuable antiseptic. It is quite as potent as chloride of zinc or carbolic acid, and is at the same time non-poisonous, and devoid of unpleasant smell of every kind. These qualities will no doubt ensure its being extensively used, and at no distant date we may expect it to displace the antiseptics which are at present in vogue.

It is somewhat strange that this substance should have been so long overlooked as a possible antiseptic, and Mr. Gambee certainly deserves credit for suggesting the utilization of it for this purpose. The reason why it has been passed over is probably to be sought in its not being a waste product in any common chemical manufacture. The anhydrous chloride aluminium, which is manfactured in order to serve for the preparation of metallic aluminium, is far too costly on account of the troublesome nature of the process by which it is prepared—to wit, by passing chlorine at high temperatures over a mixture of aluminium and charcoal. By placing the anhydrous chloride of aluminium in water, it is of course converted into hydrated chloride.

The most encoomical process for the preparation of the hydrated chloride of aluminium appears to be by double decomposition between sulphate of alumina and chloride of calcium (both of which are cheap commercial products). When solutions of these two salts are mixed together, sulphate of lime is formed and appears as a precipitate, whilst the hydrated chloride of aluminium remains dissolved.

On allowing the aqueous solution to evaporate at a very gentle heat and afterwards cooling, crystals of hydrated chloride are produced. If an attempt be made to drive off the water from the hydrated chloride by the application of heat, decomposition will take place. Hydrochloric acid is evolved under these conditions, and oxychloride of aluminum is formed, and, by pushing the process, alumina is obtained as the ultimate fixed product.—*Lancet.*

